# Higher consumption of ultra-processed foods and increased likelihood of central nervous system demyelination in a case-control study of Australian adults

**DOI:** 10.1038/s41430-023-01271-1

**Published:** 2023-02-08

**Authors:** Adriana Mannino, Alison Daly, Eleanor Dunlop, Yasmine Probst, Anne-Louise Ponsonby, Ingrid A. F. van der Mei, the Ausimmune Investigator Group, Caron Chapman, Alan Coulthard, Keith Dear, Terry Dwyer, Trevor Kilpatrick, Robyn Lucas, Tony McMichael, Anne-Louise Ponsonby, Bruce Taylor, Patricia Valery, Ingrid A. F. van der Mei, David Williams, Lucinda J. Black

**Affiliations:** 1 Curtin School of Population Health, Curtin University, Perth, Western Australia Australia; 2 Faculty of Science, Medicine and Health, University of Wollongong, Wollongong, New South Wales Australia; 3 Illawarra Health and Medical Research Institute, Wollongong, New South Wales Australia; 4 Murdoch Childrens Research Institute, Royal Children’s Hospital, Melbourne, Victoria Australia; 5 Department of Neuroepidemiology, The Florey Institute of Neuroscience and Mental Health, Parkville, Victoria Australia; 6 Menzies Institute for Medical Research, University of Tasmania, Hobart, Tasmania Australia; 7 Curtin Health Innovation Research Institute, Curtin University, Perth, Western Australia Australia; 8 Barwon Health, Geelong, Victoria Australia; 9 Royal Brisbane and Women’s Hospital and the University of Queensland, Brisbane, Queensland Australia; 10 School of Public Health, University of Adelaide, Adelaide, South Australia Australia; 11 Centre for Neuroscience, University of Melbourne, Melbourne, Victoria Australia; 12 National Centre for Epidemiology and Population Health, The Australian National University, Canberra, Australian Capital Territory Australia; 13 QIMR Berghofer Medical Research Institute, Brisbane, Queensland Australia; 14 Hunter Health, Newcastle, New South Wales Australia

**Keywords:** Autoimmune diseases, Nutrition

## Abstract

**Background:**

Consumption of ultra-processed foods (UPFs) has been linked to risk of chronic diseases, with scant evidence in relation to multiple sclerosis (MS).

**Methods:**

We tested associations between UPF consumption and likelihood of a first clinical diagnosis of central nervous system demyelination (FCD) (267 cases, 508 controls), a common precursor to MS. We used data from the 2003–2006 Ausimmune Study and logistic regression with full propensity score matching for age, sex, region of residence, education, smoking history, body mass index, physical activity, history of infectious mononucleosis, dietary misreporting, and total energy intake.

**Results:**

Higher UPF consumption was statistically significantly associated with an increased likelihood of FCD (adjusted odds ratio = 1.08; 95% confidence interval = 1.0,1.15; *p* = 0.039), representing an 8% increase in likelihood of FCD per one energy-adjusted serving/day of UPFs.

**Conclusion:**

Higher intakes of UPF were associated with increased likelihood of FCD in this Australian cohort. Nutrition education and awareness of healthy eating patterns may benefit those at high risk of FCD.

## Introduction

Advances in food technology over the last century have accelerated the development of ultra-processed foods (UPFs) (e.g., packaged snacks, confectionery, instant/ready-to-eat meals, margarine, processed meats, pastries). UPFs are industrial formulations usually containing many ingredients not commonly used in culinary preparations; they are typically energy dense and nutrient poor [1]. A recent review showed that higher consumption of UPFs was associated with a higher risk of cardiovascular and cerebrovascular disease, depression, and all-cause mortality [2]. Using the Ausimmune Study, we have previously shown that healthy patterns of eating are associated with reduced likelihood of a first clinical diagnosis of central nervous system (CNS) demyelination (FCD) [3], a common MS precursor. Here, we tested associations between consumption of UPFs and likelihood of FCD.

## Methods

In the 2003–2006 Ausimmune Study, case participants (*n* = 282) presenting with symptoms suggestive of inflammatory CNS demyelination were notified to the study by clinicians from four regions of Australia. Between one and four controls (*n* = 558) were recruited via the Australian Electoral Roll and matched to each case, outlined below, by age (±2 years), sex and study region. Participant cases had an FCD within the study period; date of onset and symptoms were confirmed by a study neurologist [4]. The diagnoses included: a classic first demyelinating event (FDE; defined as a single, first, episode of clinical symptoms suggestive of CNS demyelination; *n* = 216); a first recognised event, but past history revealed a prior, undiagnosed event, that, on review was highly suggestive of CNS demyelination (*n* = 48); first presentation of primary progressive MS (based on neurologic assessment on study entry) (*n* = 18). Participants were aged between 18 and 59 years. Detailed methodology is outlined elsewhere [4]. Ethics approval was obtained from the Human Research Ethics Committees of the participating institutions [4]. Participants provided written informed consent.

Dietary intake in the 12 month period prior to the study interview was assessed using a food frequency questionnaire (FFQ) (*n* = 791) of 101 food and beverage items, as previously described [3]. Participants with plausible energy intakes (3000–21000 kJ/day) [5] (*n* = 775; 267 cases, 508 controls) were included in our study. We identified UPFs according to category four of the NOVA classification system, which groups foods based on amount of industrial processing [1]. For the 28 UPF items captured in the FFQ, we converted intakes in g/day to servings/day using recommended serving sizes (Supplementary Table 1).

Along with information on age, sex and region of residence (the matching variables), education, smoking history, physical activity (International Physical Activity Questionnaire) and history of infectious mononucleosis were collected from self-reported questionnaires. The study nurse measured height and weight to calculate body mass index (BMI). We created a two-category variable for dietary misreporting (normal/over-reporter *vs*. under-reporter), as previously described [3], using energy intake:basal metabolic rate and the Goldberg cut points [6].

In the sample description, we used frequency and percentage for categorical variables, mean and standard deviation for normally distributed continuous variables, and median and interquartile range for non-normally distributed continuous variables. Pearson chi square, Wilcoxon rank sum and *t*-tests were used to test differences in characteristics between cases and controls. We used the residual method to energy-adjust the servings/day of each UPF item, and investigated interactions between consumption of energy-adjusted UPFs and total energy intake, BMI and physical activity. We tested associations between reported consumption of UPFs (energy-adjusted servings/day) and likelihood of an FCD using logistic regression, with full propensity score matching [7]. Matching variables were: sex, age, study region (the same matching variables in selection of controls); education, smoking history, BMI, physical activity, history of infectious mononucleosis; dietary misreporting and total energy intake (to account for energy under-reporting and energy intake). The multivariable analysis included 734 participants (257 cases, 477 controls). We conducted a post estimation test for the overlap assumption for the matched groups.

The final model was bootstrapped (500 repetitions) with bias corrected adjustment. We used Stata 14 (StataCorp, Texas, USA), with statistical significance of *P* < 0.05.

## Results

Compared with controls, a higher percentage of cases had a history of smoking and a history of infectious mononucleosis (Table 1). We found no statistically significant interactions between consumption of energy-adjusted UPFs and total energy intake, BMI or physical activity. The overlap assumption for fully matched propensity scoring was met. Higher consumption of UPFs was significantly associated with increased likelihood of FCD (adjusted odds ratio = 1.08; 95% confidence interval = 1.00,1.15; *p* = 0.039), with an 8% increase in the likelihood of FCD per one energy-adjusted serving/day.

**Table 1:** Characteristics of participants included in the current study (*n* = 775, 267 cases, 508 controls).

	Case (*n* = 267)	Control (*n* = 508)	*P*
Sex, *n* (%)			
Male	113 (23.2)	62 (22.2)	0.757
Female	395 (76.8)	205 (77.8)	
Age in years, median (IQR)	38.5 (14.7)	39.8 (15.1)	0.123
Study region, *n* (%)			
Brisbane (27 °S)	90 (33.7)	176 (34.6)	0.089
Newcastle (33 °S)	35 (13.1)	88 (17.3)	
Geelong (37 °S)	64 (24)	134 (26.4)	
Tasmania (43 °S)	78 (29.2)	110 (21.7)	
Education, *n* (%)			
Year 10 or below	66 (24.8)	165 (32.5)	0.065
Year 11 or 12	53 (19.9)	73 (14.4)	
TAFE/Diploma	80 (30.1)	139 (27.4)	
University	67 (25.2)	131 (25.8)	
Smoking history, *n* (%)			
Smoked at some time	163 (61.3)	266 (52.5)	0.019
Never smoked	103 (38.7)	241 (47.5)	
Body mass index, median (IQR)	25.9 (7.6)	25.7 (7.4)	0.848
Physical activity (METs), median (IQR)	2034 (3639)	1940 (3137)	0.596
History of infectious mononucleosis, *n* (%)			
Yes	73 (27.4)	83 (16.3)	<0.0001
No	175 (65.8)	404 (79.5)	
Do not know	18 (6.8)	21 (4.1)	
Dietary misreporting, *n* (%)			
Under-reporter	73 (27.4)	176 (22)	0.091
Normal/over-reporter	193 (72.6)	394 (78)	
Ultra-processed foods (servings/day), median (IQR)	6.2 (4.3)	5.5 (4.4)	0.889

*IQR* interquartile range, *TAFE* Technical and Further Education, *SD* standard deviation; *MET* metabolic equivalent of task.

The following variables had missing data: education (1 case); smoking history (1 case, 1 control); body mass index (1 case, 3 controls); history of infectious mononucleosis (1 case); dietary misreporting (1 case, 3 controls).

## Discussion

To our knowledge, this is the first study to investigate consumption of UPFs and likelihood of FCD, or of MS. Higher consumption of UPFs was statistically significantly associated with increased likelihood of FCD. This complements our previous findings that a more healthy pattern of eating (high in poultry, fish, eggs, vegetables, legumes) was associated with lower likelihood of FCD [3].

Based on national survey data, dietary energy density in the Australian population increased between 1995 and 2012 [8], and UPFs contributed 42% of total energy intake in 2011–2012 [9]. The increasing amount of UPFs in the food supply has led to the displacement of healthier patterns of eating that are based on fresh and minimally processed whole foods [9], which is a potential explanation for the findings of our study. Another potential mechanism is the link between consumption of UPFs, gut microbiota and neurodegenerative diseases. High consumption of UPFs, which promotes gut dysbiosis, may induce the production of proinflammatory cytokines that in turn promote neuroinflammation and neurodegeneration [10]. Furthermore, consuming UPFs may increase exposure to synthetic chemicals, such as phthalates, which are used in packaging and production, and have been associated with wide-ranging adverse health outcomes [11].

A strength of our study was using data from the Ausimmune Study, one of the largest studies of early MS, allowing us to control for various potential confounders. While we used an established FFQ, administered soon after FCD, the tool was not specifically designed to collect data on UPFs nor to account for the changes occurring in the food supply. Hence, some UPFs, such as sugar-sweetened beverages, energy bars, and some mass-produced breads, were not captured, yet are known to be consumed in large amounts by the Australian population [9]. Some food items were captured in the FFQ as groups, and included foods that were not ultra-processed (e.g., honey and syrups were included with jam and marmalade; tomato paste and dried tomatoes were included with tomato sauce), highlighting the complexity of assigning a single value to a group of often diverse food types. As the study population was predominately Caucasian, the findings may not be generalisable to other populations.

Our results show that higher consumption of UPFs was associated with an increased likelihood of FCD in this Australian population. Increased awareness about healthy eating, along with improved nutrition education, may help those at risk of FCD, or MS, to follow healthier patterns of eating.

## Supplementary information

Supplementary Table 1

## Data Availability

The data analysed in this study were obtained from the Ausimmune Study. The data can be made available for analysis with a collaborative agreement with the Ausimmune Investigator Group. Requests to access these datasets should be directed to Professor Anne-Louise Ponsonby, annelouise.ponsonby@florey.edu.au.

## References

[1] Monteiro CA, Cannon G, Levy RB, Moubarac JC, Louzada ML, Rauber F (2019). Ultra-processed foods: what they are and how to identify them. Public Health Nutr.

[2] Pagliai G, Dinu M, Madarena MP, Bonaccio M, Iacoviello L, Sofi F (2021). Consumption of ultra-processed foods and health status: a systematic review and meta-analysis. Br J Nutr.

[3] Black LJ, Rowley C, Sherriff J, Pereira G, Ponsonby A-L, Ausimmune Investigator Group, (2019). A healthy dietary pattern associates with a lower risk of a first clinical diagnosis of central nervous system demyelination. Mult Scler.

[4] Lucas RM, Ponsonby AL, McMichael A, van Der Mei I, Chapman C, Coulthard A (2007). Observational analytic studies in multiple sclerosis: controlling bias through study design and conduct. The Australian multicentre study of environment and immune function. Mult Scler.

[5] Ambrosini GL, Van Roosbroeck SAH, Mackerras D, Fritschi L, De Klerk NH, Musk AW (2003). Reliability of ten-year dietary recall: implications for cancer research. J Nutr.

[6] Black AE (2000). The sensitivity and specificity of the Goldberg cut-off for EI:BMR for identifying diet reports of poor validity. Eur J Clin Nutr.

[7] Austin PC, Stuart EA (2017). Estimating the effect of treatment on binary outcomes using full matching on the propensity score. Stat Methods Med Res.

[8] Grech AL, Rangan A, Allman-Farinelli M (2017). Dietary energy density in the australian adult population from national nutrition surveys 1995 to 2012. J Acad Nutr Diet.

[9] Machado PP, Steele EM, Levy RB, Sui Z, Rangan A, Woods J (2019). Ultra-processed foods and recommended intake levels of nutrients linked to non-communicable diseases in Australia: evidence from a nationally representative cross-sectional study. BMJ open.

[10] Martínez Leo EE, Segura Campos MR (2020). Effect of ultra-processed diet on gut microbiota and thus its role in neurodegenerative diseases. Nutrition.

[11] Buckley JP, Kim H, Wong E, Rebholz CM (2019). Ultra-processed food consumption and exposure to phthalates and bisphenols in the US National Health and Nutrition Examination Survey, 2013-2014. Environ Int.

