# Supplementary material for: Higher consumption of ultra-processed foods and increased likelihood of central nervous system demyelination in a case-control study of Australian adults
Source: Eur J Clin Nutr. 2023 Feb 8;77(5):611–4. doi: 10.1038/s41430-023-01271-1 (PMC10169648; doi:10.1038/s41430-023-01271-1)
Supplement: Supplementary file 1 — Supplementary Table 1 [file 41430_2023_1271_MOESM1_ESM.docx]

Supplementary Table 1 Recommended serving sizes of the ultra-processed food and beverage items reported in the food frequency questionnaire

| **Food or beverage item** | **Grams per serving** |
| --- | --- |
| All-bran^™^ | 30 g |
| Bacon | 50 g |
| Margarine blends | 20 g |
| Sultana Bran^™^, FibrePlus^™^, Bran flakes^™^ | 30 g |
| Cakes, sweet pies, tarts and other sweet pastries | 40 g |
| Chocolate | 25 g |
| Cornflakes, Nutrigrain^™^, Special K^™^ | 30 g |
| Corn chips, potato crisps, Twisties^™^, etc. | 30 g |
| Crackers, crispbreads, dry biscuits | 30 g |
| Flavoured milk drink (cocoa, Milo^™^, etc.) | 250 g^1^ |
| Ham | 55 g |
| Hamburger with a bun | 55 g^1^ |
| Hot chips | 60 g |
| Ice-cream | 75 g |
| Jam or marmalade | 60 g |
| Luncheon meats or salami | 55 g |
| Margarine of any kind | 20 g |
| Meat pies, pasties, quiche and other savoury pastries | 60 g |
| Monounsaturated margarine | 20 g |
| Muesli | 30 g |
| Pizza | 62 g^1^ |
| Polyunsaturated margarine | 20 g |
| Sausages or frankfurters | 55 g |
| Sweet biscuits | 30 g^1^ |
| Tomato sauce | 15 g^2^ |
| Vegemite^™^, Marmite^™^ or Promite^™^ | 5 g^2^ |
| Weetbix^™^, Vita Brits^™^, Weeties^™^ | 30 g |
| White bread^4^ | 40 g |

Except where indicated, serving sizes were from the Australian Healthy Eating Guidelines (National Health and Medical Research Council. Australian Dietary Guidelines. Canberra, ACT: Australian Government, 2015. Available from: https://www.eatforhealth.gov.au/guidelines)

^1^ Food Standards Australia New Zealand (FSANZ). Australian Food Composition Database - Release 1. Canberra, ACT: FSANZ, 2019. Available from: https://www.foodstandards.gov.au/science/monitoringnutrients/afcd/pages/default.aspx

^2^ Information from product label.
